# Human Corneal Expression of SLC4A11, a Gene Mutated in Endothelial Corneal Dystrophies

**DOI:** 10.1038/s41598-019-46094-y

**Published:** 2019-07-04

**Authors:** Darpan Malhotra, Sampath K. Loganathan, Anthony M. Chiu, Chris M. Lukowski, Joseph R. Casey

**Affiliations:** 1 Department of Biochemistry, Membrane Protein Disease Research Group, University of Alberta, Edmonton, Alberta T6G 2H7 Canada; 2 Present Address: Lunenfeld-Tanenbaum Research Institute, Toronto, Ontario M5G 1X5 Canada

**Keywords:** Membrane proteins, Corneal diseases

## Abstract

Two blinding corneal dystrophies, pediatric-onset congenital hereditary endothelial dystrophy (CHED) and some cases of late-onset Fuchs endothelial corneal dystrophy (FECD), are caused by SLC4A11 mutations. Three N-terminal SLC4A11 variants: v1, v2 and v3 are expressed in humans. We set out to determine which of these transcripts and what translated products, are present in corneal endothelium as these would be most relevant for CHED and FECD studies. Reverse transcription PCR (RT-PCR) and quantitative RT-PCR revealed only v2 and v3 mRNA in human cornea, but v2 was most abundant. Immunoblots probed with variant-specific antibodies revealed that v2 protein is about four times more abundant than v3 in human corneal endothelium. Bioinformatics and protein analysis using variant-specific antibodies revealed that second methionine in the open reading frame (M36) acts as translation initiation site on SLC4A11 v2 in human cornea. The v2 variants starting at M1 (v2-M1) and M36 (v2-M36) were indistinguishable in their cell surface trafficking and transport function (water flux). Structural homology models of v2-M36 and v3 suggest structural differences but their significance remains unclear. A combination of bioinformatics, RNA quantification and isoform-specific antibodies allows us to conclude that SLC4A11 variant 2 with start site M36 is predominant in corneal endothelium.

## Introduction

Cornea, the outer surface of the eye, serves as a physical barrier to protect the inner layers of the eye and contributes to refractive power in focusing the visual image^1^. Within the cornea are three major layers: outermost is the epithelium; the middle layer is the stroma, a dissolved proteoglycan and collagen layer, sparsely populated by keratocytes; innermost is the endothelium, a monolayer of endothelial cells, which rest on their basement membrane, the Descemet’s membrane (DM). Because of the stroma’s high solute concentration, there is a strong drive for osmotic fluid accumulation from the aqueous humor, across the endothelium. The endothelial layer’s principal role is to remove this fluid through a “pumping” mechanism, which reabsorbs fluid into the aqueous humor^2^.

Defects in the endothelial “pump” give rise to stromal fluid accumulation, significantly thickening the layer. The edematous stroma blurs vision, significantly compromising visual acuity. Defects in the corneal endothelium give rise to posterior endothelial corneal dystrophies (ECD). Dominantly inherited, Fuchs endothelial corneal dystrophy (FECD) is most common, with a lifetime incidence of about 4% and disease onset typically over age 40^1^. FECD is responsible for 24% of corneal transplantation procedures in United States^3^. FECD is genetically heterogenous and caused by alterations in multiple genes including, *SLC4A11*^4^, *COL8A2*^5^, *TCF8*^6^, *AGBL1*^7^, *LOXHD1*^8^, *KANK4*^9^, *LAMC1*^9^ and *ATPB1*^9^, although trinucleotide repeat expansion in *TCF4*^10^, is estimated to be responsible for about 70% of cases. Recessively-inherited, childhood-onset congenital hereditary endothelial dystrophy (CHED) arises only from SLC4A11 mutations^1,11–16^. Finally, SLC4A11 mutations also cause Harboyan syndrome (HS), characterized by childhood endothelial dystrophy and progressive sensorineuronal deafness^17^, although recently HS has been interpreted as a manifestation of CHED^18^.

SLC4A11 is an integral membrane protein, with a 41 kDa cytoplasmic domain and a 57 kDa membrane domain. Consistent with an important role in the endothelial cell fluid “pump”, SLC4A11 facilitates transmembrane water movement^19,20^, Na^+^/OH^−^ co-transport^21,22^, Na^+^-independent H^+^ (OH^−^) transport^23,24^, and NH_3_ transport^25,26^. Although plant SLC4A11 orthologs can transport borate^27^, the report that human SLC4A11 can^28^, has been disputed^21,22^. Most disease alleles of SLC4A11 result in protein misfolding and protein retention in the endoplasmic reticulum^4,15,29^, but some affect the protein’s transport function^19,23,30^.

The genome database reveals three N-terminal variants of human SLC4A11: 918 amino acid splice form 1 (NCBI Reference Sequence: NP_001167561.1), 891 amino acid splice form 2 (NP_114423) and 875 amino acid splice form 3 (NP_001167560). These variants have also been respectively called SLC4A11-A, -B and –C^23^. Differences in the three sequence variants arise at the N-terminus within the first 20–60 amino acids. One RT-PCR analysis of RNA from corneal endothelium suggested that only splice form 3 was present^23^. Studies of recombinant SLC4A11 are ongoing, working to understand SLC4A11 function and ultimately to ameliorate corneal dystrophy symptoms associated with defective SLC4A11. To date, all earlier studies have explored the function of recombinant splice form 2^4,15,19–22,25,31–34^.

Definitively establishing the form of SLC4A11 protein in human cornea is critical to guide relevant future studies of the protein. Approximately 60 point mutations are spread throughout the two domains of SLC4A11^4,11–15,17,32,35–39^. SLC4A11 has a large N-terminal cytoplasmic domain, which has a role in SLC4A11 solute transport function^31^. The three N-terminal variants may thus affect the structure and/or function of the N-terminal cytoplasmic domain, with potential impact on transport activity. This underscores the need to identify the SLC4A11 isoform(s) present in human cornea. Accordingly, we analyzed intact human corneas and isolated corneal endothelium (the innermost layer of endothelial cells peeled off along with their basement membrane, the DM). We performed RT-PCR and real-time quantitative PCR of RNA from intact human cornea and isolated corneal endothelium. We developed antibodies able to detect variant 2 initiating translation at two possible start Methionine (Met) residues, and variant 3. We further characterized the cell surface trafficking, transport functions and structural differences of SLC4A11 variants.

## Results

### Bioinformatic analyses of human SLC4A11

**Figure 1:**
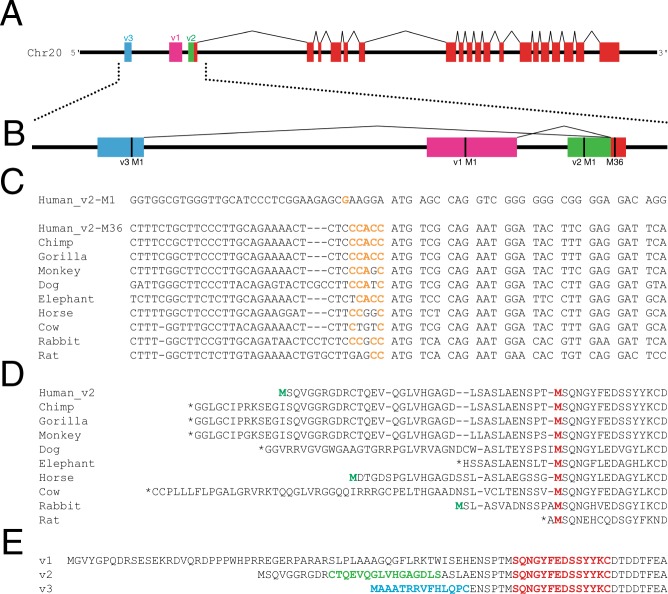
Bioinformatic analysis of human SLC4A11. (**A**) cDNA sequences of known human SLC4A11 transcripts were mapped onto a 15 kb segment of Human chromosome 20 genomic DNA to show transcript structure (coloured boxes), connected by introns (black lines). Each transcript variant (v1, v2 and v3) has a unique starting exon which results in unique N-terminal regions of the predicted protein and a common core region (red). (**B**) Magnified view of the 5′ end of the SLC4A11 gene. Each variant has a predicted start codon (M1; vertical black line). M36 (numbering based on predicted sequence of v2) marks the start of the common coding sequence for all transcripts. (**C**) Alignment of DNA sequences surrounding the predicted start codons in SLC4A11 v2 for multiple species. Kozak translational start sequence efficiency^40^ is indicated with bases matching the consensus shown in orange. For human SLC4A11, Kozak analysis is indicated for v2 start at Met 1 and 36 (v2-M1 and v2-M36). (**D**) Alignment of amino acid sequences for v2 SLC4A11 for the indicated mammals. Red Methionine (M) residue indicates the position corresponding to human v2-M36. Green Met indicates in frame Met upstream of v2-M36. Stop codons are indicated (*). (**E**) Amino acid sequences of SLC4A11 v1-v3 are aligned with sequences of peptides used to generate splice form-selective antibodies highlighted in red: SLC4A11-common, green: SLC4A11-v2-M1 and blue: SLC4A11-v3.

To begin to examine what forms of SLC4A11 are expressed in human cornea, we performed bioinformatic analysis (Fig. 1). Three variant transcripts of human SLC4A11 have been identified by cDNA sequencing efforts: variant 1 (v1), variant 2 (v2) and variant 3 (v3). These share 18 common exons and differ only in the sequence of their first exon (Fig. 1A). According to the cDNA sequences deposited into genome databases, transcript v1 and v3 splice into the middle of the first exon of v2. The genetic basis for this observation is unclear as splicing typically occurs at intron-exon boundaries, not in the middle of an exon. Nonetheless, sequences of transcripts are consistent with the splicing diagram presented (Fig. 1B).

We next examined coding sequences for the equivalent of v2 amongst mammalian species (Fig. 1C). In the case of human v2, we aligned the sequences around the first (M1) and second (M36) potential start methionine residues. Nucleotide sequence conservation was clearly much stronger at the start codon for Met36 than the upstream M1, which was even more evident at the amino acid level (Fig. 1D). This led us to consider whether M36 might act as the translational start site for the protein. The nucleotide sequence surrounding the start codon, the Kozak consensus sequence, is important in establishing translational start efficiency^40^. We thus mapped the translational start efficiency of nucleotides surrounding human SLC4A11 variant 2 at M1 (v2-M1) and M36 (v2-M36), as well as the other mammalian sequences (Fig. 1C). For translational start at v2-M1, only one of six nucleotides match optimal Kozak consensus (Fig. 1C). Yet, v2-M36, shows 5/6 upstream nucleotides as agreeing with consensus for efficient translational start. Moreover, all analyzed mammalian sequences revealed adequate-strong prediction around the ATG corresponding to human v2-M36 as translational start site, on the basis of Kozak consensus, GCCACCATG.

We next aligned amino acid sequences predicted for SLC4A11 v2 transcripts in ten mammals, including human (Fig. 1D). The alignment shows a high degree of amino acid identity starting at M36 of human SLC4A11. Moreover, only a small proportion of mammals (2/9 shown) had a potential start Met codon upstream of the position corresponding to human Met36. Together these observations suggest that in humans SLC4A11 v2 translational start occurs at the second Met in the sequence, Met36.

To determine the form of SLC4A11 expressed in cornea, antibodies able to detect specific sequences of SLC4A11 were prepared. Synthetic peptides were synthesized and used to immunize rabbits. Antibody SLC4A11-common was raised against a sequence immediately after the v2-M36, thus detecting a region common to all SLC4A11 variants (Fig. 1E, red). Antibody SLC4A11-v2-M1 was raised against a sequence uniquely present in the region between M1 and M36 of v2 (Fig. 1E, green). Finally, antibody SLC4A11-v3 was raised against a peptide corresponding to the unique region of v3 (Fig. 1E, blue).

### Expression of SLC4A11 transcripts in human cornea

To assess the SLC4A11 variants expressed in human cornea, we obtained human corneas deemed unsuitable for transplant. Whole cornea contains three predominant cell types. There is an outer epithelial cell layer, a stromal layer largely acellular, but containing some keratocytes, and an inner endothelial cell layer. RNA prepared from whole cornea thus represents the contribution of at least three cell types. Corneal dystrophies arise from defects in the endothelial layer, so we also prepared RNA from isolated corneal endothelium, which was peeled-off from the corneas with the DM.

**Figure 2:**
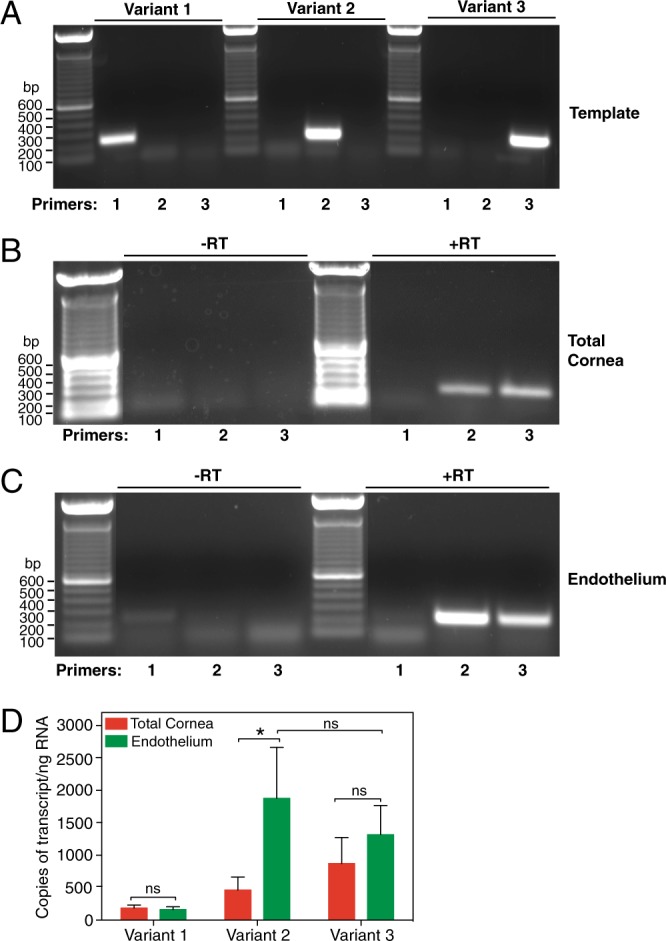
Expression of SLC4A11 transcripts in human cornea. (**A**) Plasmid-cloned SLC4A11 v1, v2 and v3 were each used as a template with specific forward primers for v1, v2 and v3, respectively and a common reverse primer. PCR reaction was set up for 40 cycles, and total reaction mixture was loaded on a 1% agarose gel. (**B,C**) RNA isolated from total cornea and micro-dissected endothelial layer were subjected to reverse transcription reaction with (+RT) or without (−RT) reverse transcriptase enzyme. PCR was performed using cDNA template generated from (**B**) total cornea and (**C**) isolated endothelium, with forward primers corresponding to v1, v2 and v3 along with common reverse primer. Expected amplicon sizes were 244, 233 and 233 bp for v1, v2 and v3, respectively. (**D**) qPCR was performed on cDNA from corneal endothelium and total cornea using iQ SYBR green mix along with forward and reverse primers for v1, v2 and v3 and GAPDH. Reactions were carried out for 40 cycles and fluorescence was recorded at each elongation step. Standard curve was constructed for each variant by using respective synthetic templates in the range of 2.5–2.5 × 10^4^ copies and plotting Log_10_ (template copies) against their C_T_ values. The sample C_T_ values were corrected to GAPDH for every experiment and number of copies of each variant was calculated, using their respective standard curves. Background (-RT control) values were subtracted from the samples. The average number of copies per ng RNA were plotted for each variant in corneal endothelium and total cornea. Data are presented as mean ± SEM from three biological replicates (Two-way ANOVA and Sidak’s multiple comparison test). *p = 0.02, ns = not significant.

RT-PCR was performed using primer pairs (see Supplementary Table S1) producing products spanning intron-exon boundaries to avoid background arising from genomic DNA contamination of RNA samples. Primers were designed to amplify each of the three reported SLC4A11 mRNA variants, which differ in their first exon as shown in Fig. 1A. Products were observed using agarose gel electrophoresis. To verify the ability of PCR primers to detect all SLC4A11 variants, PCR was performed on SLC4A11 v1, v2 and v3 cDNA cloned into a plasmid vector. v2 and v3 were expression constructs cloned into pcDNA3.1. In the case of v1, synthetic DNA including the entire v1 PCR amplicon was cloned into pJET1.2 plasmid to use as PCR template. PCR was carried out using the same molar amount of plasmid template for each variant, and with the specific PCR primer sets for the three variants on each template. Using cloned templates, v1 PCR product was obtained with a similar intensity to the bands found for the v2 and v3 amplicons (Fig. 2A). Thus, v1 reverse transcription product would be detected if its expression in human cornea were similar to v2 or v3. Specificity of RT-PCR was confirmed by the presence of PCR product only when PCR was performed with the primer pair designed for each variant, but not when tested on the other templates (Fig. 2A).

Reverse transcription reactions were performed with and without reverse transcriptase enzyme. RT-PCR of samples produced without reverse transcriptase did not produce any product, indicating that bands observed arose from reverse transcription of RNA (Fig. 2B,C). Using RNA from whole cornea, bands of the expected amplicon size were observed using v2 and v3-specific primers, but no band was observed for v1 (Fig. 2B). RT-PCR of human corneal endothelium RNA also revealed presence of v2 and v3 but no band was observed for v1 (Fig. 2C).

To measure the expression level of each variant in human cornea, we performed quantitative RT-PCR with cDNA from total cornea and isolated corneal endothelium from three different donors. Amounts of SLC4A11 RNA were normalized to GAPDH RNA level in each sample. Cloned cDNA for each variant (2.5–2.5 × 10^4^ copies) was used to construct standard curves to enable comparisons of numbers of transcript copies present in each sample, for each SLC4A11 variant (Fig. 2D). This approach confirmed that there is little or no v1 expressed in human cornea. Expression of v2 was enriched in corneal endothelium, in comparison to total cornea. In contrast, there was no significant difference in v3 abundance between total cornea and endothelium. In endothelium samples, no significant difference was observed between v2 and v3 abundance. v2 expression in endothelium, however, was higher in each of the three human corneas analyzed when compared to v3 (not shown). Expression level differences between patients, however, led to standard error values, that failed to reach significance when values from the three corneas were pooled.

### SLC4A11 v2 and v3 protein expression in human corneal endothelium

**Figure 3:**
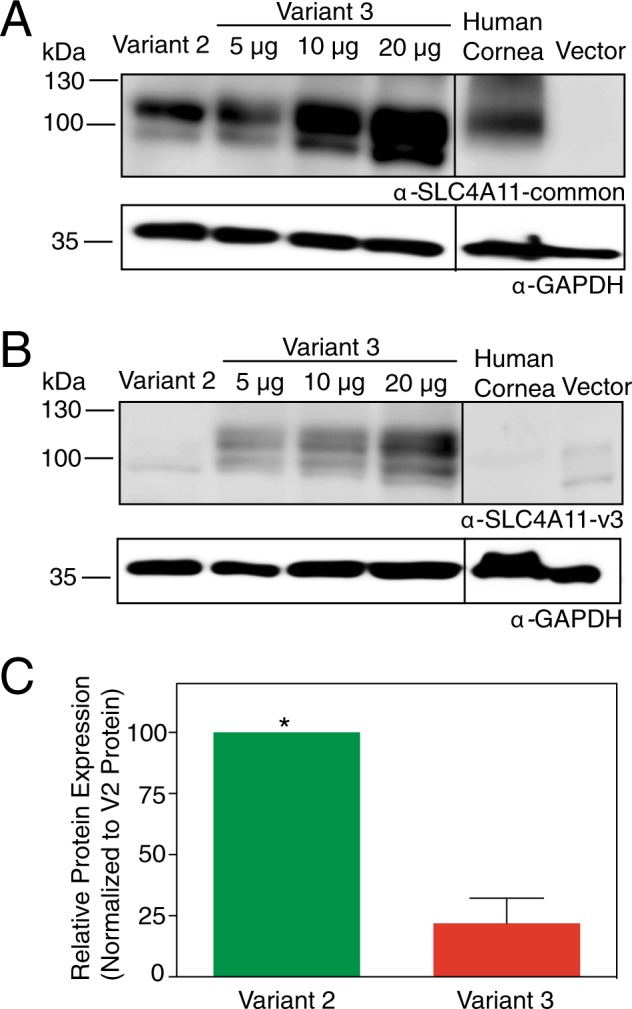
SLC4A11 v2 and v3 expression in human corneal endothelium. Corneal endothelium was micro-dissected from six human corneas. Protein lysates from pooled human corneal endothelium (20 µg protein) along with HEK293 cells transfected with cDNA encoding SLC4A11 v2 (10 µg protein lysate), v3 (5, 10, 20 µg protein lysates, indicated) and empty vector (20 µg protein lysate) were processed for immunoblots. Blots were probed with (**A**) SLC4A11-common antibody and (**B**) SLC4A11-v3 antibody, along with GAPDH antibody as a loading control. (**C**) Densitometry was performed to quantify band intensities. Detection ratio was established between SLC4A11-common antibody and SLC4A11-v3 antibody (see Supplementary Fig. S1). Data from each trial were normalized to SLC4A11 v2 abundance. Data represent mean ± SEM from three replicates (Two-tailed t-test). *p = 0.005 when compared to v3. Note that intervening lanes to the left of the human cornea lane in (**A**) and (**B**) have been removed and are marked by the black line.

To examine the expression of the translation products of these transcripts, we generated two antibodies against synthetic peptides corresponding to the SLC4A11 amino acid sequences as shown in Fig. 1E. Anti-SLC4A11-common antibody detects an epitope common to all three variants of SLC4A11 and anti-SLC4A11-v3 antibody detects the unique region in v3. On immunoblots, SLC4A11-common antibody detected HEK293 cell-expressed SLC4A11 v2 as well as variant 3 (Fig. 3A). Specificity of the common antibody was indicated by the absence of signal in vector-transfected cells. Similarly, anti-v3 antibody did not detect v2, although a faint non-specific band was visible at a molecular weight lower than v2 in transfected HEK293 cells. Band sizes are consistent with their expected sizes as shown in Fig. 1.

To quantify the abundance of v2 and v3 SLC4A11 in human corneal endothelium, corneal endothelial lysates pooled from six human corneas were processed on immunoblots. These samples were probed with the antibodies: anti-SLC4A11-common and anti-SLC4A11-v3. Both anti-SLC4A11-common and anti-SLC4A11-v3 revealed immunoreactive material consistent with SLC4A11 (Fig. 3A,B). To quantify the absolute levels of v2 and v3 protein abundance in corneal endothelium, the following approach was used. Anti-common detected both v2 and v3 proteins since they share the epitope for the antibody. Conversely, anti-v3 detected only v3 protein since the epitope is unique to v3. We reasoned that the signal intensity observed for anti-common antibody represents the sum of v2 and v3. We performed an experiment in which varied amounts of recombinant v3 SLC4A11 protein were detected with anti-SLC4A11-common and anti-SLC4A11-v3 antibodies (Fig. 3A,B). This enabled calibration of the efficiency of v3 protein detection by the two antibodies (see Supplementary Fig. S1). Using this detection ratio, we converted the v3 signal from human endothelial cell lysate probed with anti-v3 antibody, to the amount of signal that would arise using the anti-common antibody. In this way, amount of v2 in lysate = total detected by anti-common antibody – (v3 protein detected by anti-v3 antibody x detection ratio). From this analysis, v2 is about four times more abundant than v3 in human corneal endothelium (Fig. 3C).

### Identification of SLC4A11 v2 translation start site in total human corneal lysates

**Figure 4:**
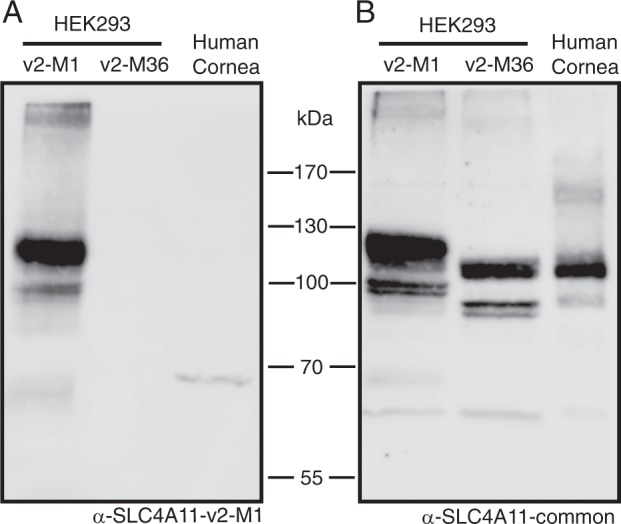
Identification of SLC4A11 v2 translation start site in total human corneal lysates. HEK293 cells were transfected with cDNA encoding: SLC4A11 v2 starting at Met1 (SLC4A11 v2-M1) or SLC4A11 v2 starting at Met36 (SLC4A11 v2-M36). Whole human cornea samples were flash frozen with liquid nitrogen, crushed, solubilized and then combined with 2X SDS-Page sample buffer. Samples were electrophoresed on 7.5% acrylamide gels. Corresponding immunoblots were probed with antibodies (**A**) Anti-SLC4A11-v2-M1 and (**B**) Anti-SLC4A11-common.

Bioinformatic analyses suggested the second methionine (M36) downstream of M1 as a potential start site on SLC4A11 v2 as shown in Fig. 1C,D. To determine whether the translational product of SLC4A11 v2 starts at M1 or M36 in the human cornea, we generated an antibody called anti-SLC4A11-v2-M1 that detects “long” form of SLC4A11 v2 starting at M1. Anti-SLC4A11-v2-M1 was raised against a peptide corresponding to a specific region up stream of M36 as shown in Fig. 1E (green). Lysates were prepared from human cornea and from HEK293 cells transfected with cDNA encoding v2-M1 or v2-M36 SLC4A11. These samples were probed on immunoblots with the antibodies: anti-SLC4A11-v2M1 and anti-SLC4A11-common. While anti-SLC4A11-v2M1 antibody only detected the HEK293 cell-expressed SLC4A11 v2 with the translation start site at M1 (Fig. 4A), SLC4A11-common antibody detected SLC4A11 v2 with translational start at M1 and M36 (Fig. 4B). Band sizes are consistent with the differences in their expected sizes as shown in Fig. 1. Specificity of the antibody was confirmed in analysis of lysates from non-transfected cells (not shown). Importantly, the SLC4A11-common antibody detected a band in human corneal lysates, migrating at a position consistent with v2-M36 (Fig. 4B), while anti-SLC4A11-v2-M1 antibody did not detect immunoreactivity in corneal cell lysates (Fig. 4A). This indicates that the SLC4A11 v2 translation start site in the human cornea is at M36 and is consistent with bioinformatic analyses, which supported M36 as translational start site.

### Plasma membrane localization of SLC4A11 v2-M1 and v2-M36

Until now, SLC4A11 v2 with M1 start site has been the focus of studies (see Supplementary Table S2)^4,15,19–22,25,31–34,41–48^. Data presented here indicate that for v2, M36, not M1, is the translational start site. The question thus arises: are these earlier reports valid? Do the properties of SLC4A11 differ depending on translational start site? We, therefore, compared the ability of these SLC4A11 variants to traffic to the cell surface and to facilitate transport function at the cell surface.

**Figure 5:**
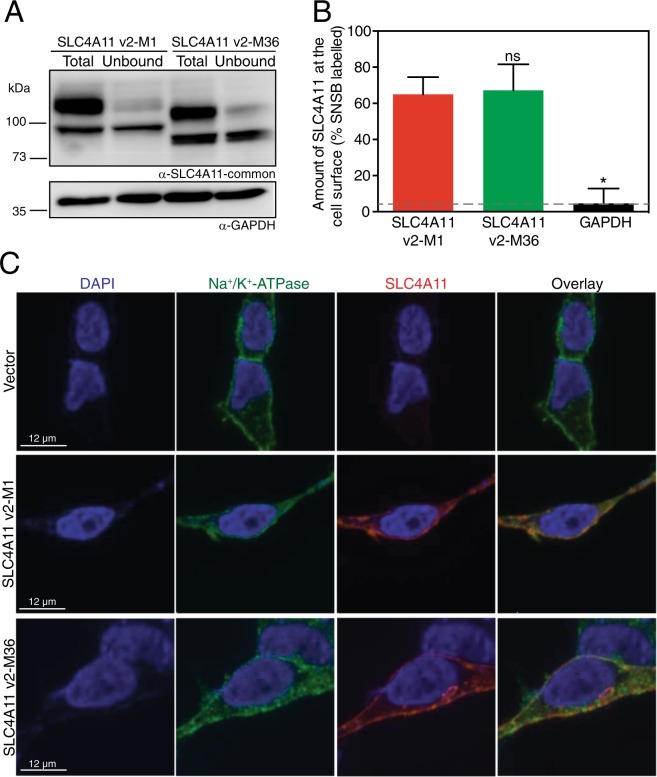
Plasma membrane localization of SLC4A11 v2-M1 and v2-M36. HEK293 cells were transfected with cDNA encoding SLC4A11 v2-M1 or SLC4A11 v2-M36 as shown. (**A**) Cells were labeled with membrane-impermeant Sulfo-NHS-SS-biotin (SNSB) and the cell lysates were divided into two equal fractions. One was incubated with streptavidin resin to remove biotinylated proteins. The unbound fraction (U) and the total cell lysate (T) were probed with SLC4A11-common antibody on immunoblots. GAPDH was used as an internal control. (**B**) The fraction of SLC4A11 and GAPDH labeled by SNSB was calculated. Dashed line indicates the background for this assay, as indicated by labeling of GAPDH. Data represent mean ± SEM from five replicates (One-way ANOVA with Sidak’s multiple comparisons test). *p < 0.0001 when compared to SLC4A11 v2-M1, ns = not significant. (**C**) Cells were processed for immunofluorescence and images were collected by confocal microscopy (scale bars indicated in each row). Nuclei were detected with DAPI staining (blue). SLC4A11 was detected with SLC4A11-common antibody and chicken anti-rabbit IgG conjugated with Alexa Fluor 594 (red). Plasma membrane marker, Na^+^/K^+^-ATPase was detected with anti-Na^+^/K^+^-ATPase and chicken anti-mouse Alexa Fluor 488 (green). Overlay represents merged images from all three channels.

cDNA encoding SLC4A11 v2-M1 or v2-M36 were expressed in HEK293 cells. Cells were treated with membrane impermeant biotinylating reagent to measure the fraction of SLC4A11 at the cells surface. Total cell lysates were incubated with streptavidin resin to remove biotinylated protein, allowing the fraction of SLC4A11 biotinylated to be assessed from the difference between total and unbound (Fig. 5A). Examining the migration position of SLC4A11 v2-M1 and v2-M36 in total lysates reveals a clear difference in migration position (Fig. 5A), which indicates that in cells transfected with v2-M1 construct, M1 is preferentially used as translational start site over M36. Thus, earlier reports of SLC4A11 studied the protein starting at the M1 of variant 2, which does not exist in the cornea.

The presence of two immune-reactive bands for SLC4A11 expressed in HEK293 cells has previously been attributed to an upper, mature-glycosylated form predominately present at the cell surface and a lower molecular weight form, receiving core glycosylation and associated with endoplasmic reticulum retention^33^. Cell surface biotinylation assays revealed no significant difference in cell surface trafficking of SLC4A11 v2 with M1 or M36 start site (Fig. 5B).

Plasma membrane localization of v2-M1 and v2-M36 SLC4A11 was further assessed by confocal immunofluorescence (Fig. 5C). Endogenous Na^+^-K^+^-ATPase was used as a plasma membrane marker. Indeed, pericellular staining was observed for Na^+^-K^+^-ATPase, consistent with its expected plasma membrane localization. Specificity of the SLC4A11-common antibody (Fig. 1E) was indicated by the absence of staining in vector-transfected cells (Fig. 5C). In cells expressing v2-M1 and v2-M36, SLC4A11 was detected with the SLC4A11-common antibody with pericellular and intracellular staining, consistent with plasma membrane and endoplasmic reticulum localizations, respectively. This staining pattern supports the conclusion from biotinylation assays that SLC4A11 protein, whether initiated at M1 or M36, gives rise to protein trafficked to the cell surface to a similar degree.

### Osmotically driven water flux activity of SLC4A11 v2-M1 and v2-M36

**Figure 6:**
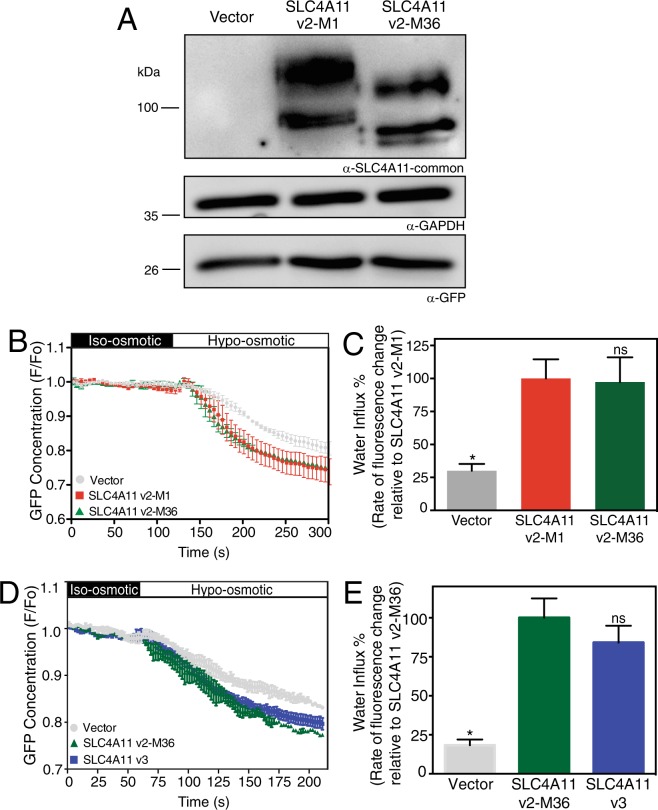
Osmotically driven water flux activity of SLC4A11 v2-M1 and v2-M36 and v3. HEK293 cells were transiently transfected with cDNA encoding eGFP along with empty vector or SLC4A11 v2-M1 or SLC4A11 v2-M36. (**A**) After performing water flux assays, cells from the same dishes were lysed and probed on immunoblots, using SLC4A11-common antibody to detect the expression of SLC4A11 variants. GAPDH and eGFP were also detected using respective antibodies. (**B**,**D**) HEK293 cells transfected with v2-M1 or v2-M36 or v3 cDNA were perfused alternately with iso-osmotic (black bar) and hypo-osmotic (white bar) media. eGFP fluorescence (F) was normalized to F averaged for time 0–120 or 0–60 seconds (F_0_) and F/F_0_ plotted. (**C**,**E**) Rate of fluorescence change (a surrogate for cell volume change) was calculated. Data represent mean ± SEM from 4–8 independent coverslips with 12–18 cells measured per coverslip (One-way ANOVA with Sidak’s multiple comparisons test) *p < 0.0001 when compared to SLC4A11 v2-M1 or SLC4A11 v2-M36, ns = not significant.

A role of SLC4A11 N-terminal cytoplasmic domain in the transport function of the protein has been suggested^31^. Thus, variations at the SLC4A11 N-terminus might affect the protein’s membrane transport function. SLC4A11 mediates accumulation of water into cells when exposed to hypo-osmotic challenge, whereas the cell surface trafficked mutant SLC4A11 R125H does not^19^. To assess water flux function, SLC4A11 N-terminal start site variants, v2-M1 and v2-M36 were expressed in HEK293 cells. Cells were co-transfected with enhanced green fluorescent protein (eGFP). Immunoblots of cells used for water flux assays revealed similar levels of SLC4A11 expression for the two variants and as expected v2-M36 SLC4A11 migrated at a lower molecular weight that v2-M1 (Fig. 6A). Similar GAPDH intensities across samples suggests that same amount of total cell lysate was loaded for all samples. Cell lysates also expressed similar levels of transfected eGFP (Fig. 6A).

To assess the capacity of SLC4A11 variants to mediate a water flux, transfected cells were placed on a perfusion chamber on the stage of a confocal microscope. Cells were initially perfused with iso-osmotic medium, followed by hypo-osmotic medium. The level of green fluorescence in a region of interest of the cytosol was monitored, as a surrogate for cell volume (Fig. 6B). Exposure to hypo-osmotic medium induced cell volume increase, as indicated by a dilution of eGFP fluorescence intensity. The initial rate of cell volume increase (decrease of eGFP fluorescence) was quantified following shift to hypo-osmotic medium (Fig. 6C). Vector-transfected cells swelled at a rate significantly slower than SLC4A11-transfected cells. The rate of cell swelling was, however, not significantly different between cells expressing the v2-M1 and v2-M36 forms of SLC4A11, indicating that SLC4A11 functional activity is not altered whether translation initiates at M1 or M36.

### Functional and structural differences between v2-M36-SLC4A11 and v3-SLC4A11

We next looked at the functional and structural differences between the two SLC4A11 variants of human corneal endothelium: v2-M36 and v3. We performed assays of osmotically driven water flux as described. No significant differences in water flux were found between HEK293 cells expressing v2-M36 and v3 (Fig. 6D,E), suggesting the unique amino acids in v3 do not affect transport function.

**Figure 7:**
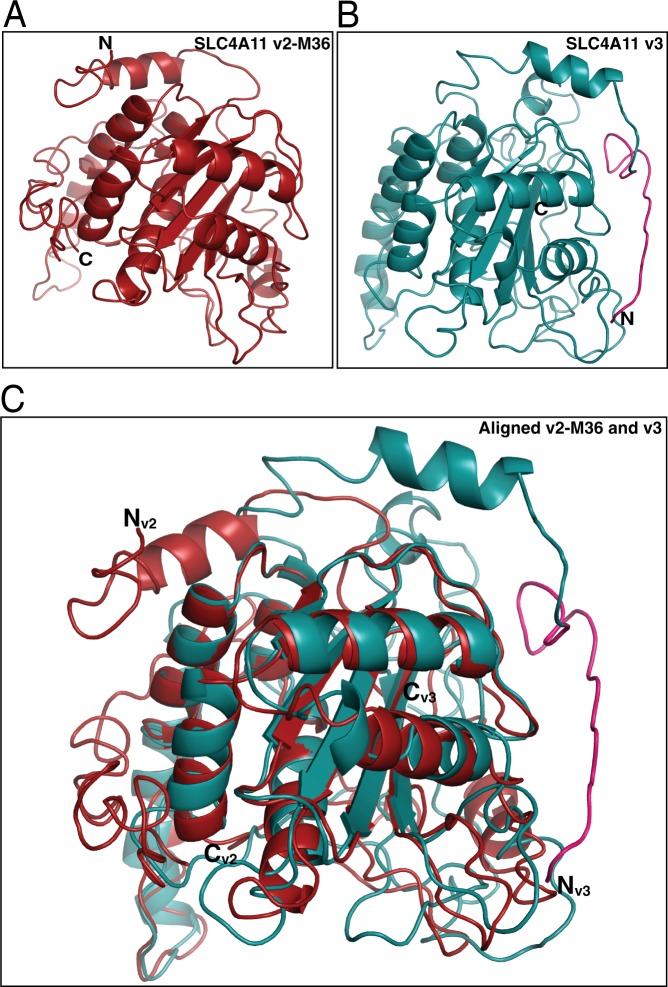
Homology model of SLC4A11 v2-M36 and SLC4A11 v3 cytoplasmic domains. Homology models of cytoplasmic domains of (**A**) SLC4A11 v2-M36 (M36-Y340) and (**B**) SLC4A11 v3 (M1-Y359) generated by I-TASSER using crystal structure of human erythrocyte Band 3 cytoplasmic domain (PDB: 1HYN) as the template. Unique N-terminal sequence of v3 is highlighted in pink. (**C**) v2-M36 and v3 models aligned in PyMOL (molecular graphics system version 2.0.7), using segments that were modelled with 100% confidence (R90-P306 for v2-M36, R109-P325 for v3). N_v2_, N_v3_ and C_v2_, C_v3_ are the N and C termini for v2-M36 and v3, respectively.

To assess the structural differences between v2-M36 and v3, we created structural homology models for the cytoplasmic domains of v2-M36-SLC4A11 (M36-Y340) and v3-SLC4A11 (M1-Y359,) using the crystal structure of human erythrocyte Band 3 (SLC4A1) cytoplasmic domain (PDB: 1HYN) as the template^49^. Earlier analysis indicated that SLC4A11 cytoplasmic domain had the same fold as Band 3^31^. Two different models were generated for each variant using Iterative Threading Assembly Refinement (I-TASSER)^50^ and Protein Homology/Analogy Recognition Engine V 2.0 (Phyre^2^)^51^. Homology models predicted through I-TASSER (Fig. 7) had estimated TM-score of 0.44 ± 0.14 (v2-M36) and 0.41 ± 0.14 (v3) and estimated RMSD of 12.0 ± 4.4 Å (v2-M36) and 12.9 ± 4.2 Å (v3). While the two structures had a common fold at their C-termini, the N-termini had significant differences (Fig. 7C). The N-terminal loops of each variant are almost 180° apart. The unique sequence at the v3 N-terminus (M1-T19, highlighted in pink), however, is surface exposed, enabling a potential role in protein-protein interaction. Homology models generated by Phyre^2^ revealed large differences in the N-terminal region of the two variants with a common conserved C-terminal fold (see Supplementary Fig. S2). Although these models highlight potential functional differences of the two variants, their N-termini were modeled with low confidence.

Computational predictions of eukaryotic phosphorylation sites in the unique N-terminal region of v3 (M1-T19) were performed using NetPhos 3.1^52^ with a cut-off score of 0.8. Threonine at position 5 (N-MAAA**T**RRVFHLQPC-C) was predicted as a potential phosphorylation site for Protein Kinase C (PKC) with high score (0.887). PKC is crucial in corneal endothelial cell proliferation and cell cycle^53^. v3-SLC4A11 could be specifically phosphorylated at Thr5 as part of corneal endothelial cell proliferative regulation.

## Discussion

This study used bioinformatic, transcriptomic and protein level analyses to identify the SLC4A11 translational product in human cornea. Bioinformatic analysis predicted SLC4A11 v2 starting at Met36 to share the highest nucleotide and amino acid sequence conservation with SLC4A11 from other mammals. RT-PCR and qRT-PCR further revealed the presence of only v2 and v3 transcripts in human cornea and the endothelium, where variant 2 is the predominant transcript in the endothelium. Protein analysis showed the predominance of v2 protein in the endothelium when compared to v3. Further, antibodies targeting v2-M1 and v2-M36 SLC4A11 confirmed the presence of SLC4A11 v2-M36 in the human cornea. Homology models of the cytoplasmic domains of v2-M36 and v3 displayed substantial differences in the N-termini of both variants with a common c-terminal fold. No differences were, however, observed in the water flux function of these variants. Data here support that v2-SLC4A11, with translation start at the transcript’s second Met is the predominant SLC4A11 protein of human corneal endothelium, although the v3 variant is present at a significant level. No difference in cell surface trafficking or transport function could be detected arising from differences in the N-terminal region of SLC4A11.

Using custom-designed antibodies, we found v2 to be four-times more abundant than v3 in the human corneal endothelium. We also found higher v2 abundance than v3 at the transcript level with negligible v1 in the cornea. Mass spectrometry, unfortunately, was unable to differentiate between v2 and v3 in the human cornea due to the absence of specific tryptic sites in the short unique N-terminal region of v3-SLC4A11 (data not shown). An earlier study found SLC4A11 v3 to be the predominant corneal endothelial SLC4A11 transcript^23^, however, the source of the endothelial mRNA used was unclear and extraction method was not described. Additionally, the earlier study did not analyze the variants at the protein level.

Variant 2 in human cornea has an alternative start site at methionine 36 (v2-M36) but most studies of SLC4A11 variant 2 have used the protein starting at methionine 1 (v2-M1) (see Supplementary Table S2). We did not find significant difference in the cell surface abundance of the v2-M36 vs v2-M1. The N-terminal cytoplasmic domain of SLC4A11, however, is required for its transport function^31^. Since we did not observe a difference of SLC4A11 water flux function between SLC4A11 v2 starting at M1 or M36, earlier functional studies of SLC4A11 v2-M1 are likely reliable. Our recent studies have used SLC4A11 v2-M36 after being identified as the relevant isoform to be studied in the cornea (see Supplementary Table S2). Only one study mentions the use of SLC4A11 v1^25^ for functional analyses (see Supplementary Fig. S2).

The presence of SLC4A11 v2-M36 and v3 in the cornea suggests unique functional roles of the two variants. Homology models of their respective cytoplasmic domains revealed large differences in the N-terminal region of the variants. The location of the unique region of v3-SLC4A11 (Fig. 7B,C) on the surface of the cytoplasmic domain is consistent with a role in protein-protein interaction. Potential phosphorylation sites in the unique region of v3 further suggests a role in protein-protein interaction and modulation by regulatory pathways. No functional differences were, however, found in osmotically driven water flux functions between the two variants. Other SLC4A11 substrates, H^+^/OH^−^/NH_3_, remain to be tested to explain the presence of two N-terminal splice variants of SLC4A11 in human cornea.

Studying the physiologically relevant form of SLC4A11 is crucial to the success investigations of SLC4A11, in particular of molecular therapeutics. Our data indicate that SLC4A11 Variant 2 starting at Met36 is the principal variant of human corneal endothelium, but variant 3 also contributes to SLC4A11’s role in corneal biology to a lesser extent.

## Materials and Methods

### Materials

Oligonucleotides were from Integrated DNA Technologies (Coralville, IA). Q5 Site-directed mutagenesis kit was from New England Biolabs (Ipswich, USA). Dulbecco’s modified Eagle’s medium (DMEM), fetal bovine serum (FBS), calf serum (CS), penicillin-streptomycin-glutamine (PSG), Chicken anti-mouse IgG conjugated with Alexa Fluor 594 and Chicken anti-rabbit Alexa Fluor 488 were from Life Technologies (Carlsbad, CA, USA). Cell culture dishes were from Sarstedt (Montreal, QC, Canada). Complete Protease Inhibitor cocktail tablets were from Roche Applied Science (Indianapolis, IN, USA). BCA Protein Assay Kit was from Pierce (Rockford, IL, USA). High Capacity Immobilized Streptavidin Agarose Resin, SuperSignal West Femto Maximum Sensitivity Substrate, Taq DNA polymerase, CloneJET PCR cloning kit, phenyl methane sulfonyl fluoride (PMSF), glass coverslips and anti-GFP antibody were from Thermo Fisher Scientific (Ottawa, ON, Canada). Poly-L-lysine was from Sigma-Aldrich (Oakville, ON, Canada). Immobilon-P PVDF membranes were from Millipore (Billerica, MA). Monoclonal antibody against glyceraldehyde-3-phosphate dehydrogenase (GAPDH) was from Santa Cruz Biotechnology (Santa Cruz, CA, USA). Monoclonal antibody against green fluorescent protein was from Thermo Fisher Scientific (Ottawa, ON, Canada). Horseradish peroxidase-conjugated sheep anti-mouse IgG was from GE Healthcare Bio-Sciences Corp. (Piscattaway, NJ, USA). ECL chemiluminescent reagent was from Perkin Elmer Life Sciences (Waltham, MA, USA). iQ SYBR green super mix was from Bio-Rad Laboratories (Hercules, CA, USA). Trizol was from Life Technologies (Carlsbad, CA, USA).

### DNA constructs

Eukaryotic-expression construct for splicing variant 2 of human SLC4A11 encoding an 891 amino acid protein (NCBI Reference Sequence: NG_017072.1), N-terminally tagged with the HA epitope (pSKL1), was reported earlier^34^. This construct encodes “long” SLC4A11, starting at presumed start codon Met 1 (SLC4A11 v2-M1). A shortened version of pSKL1, removing the first 35 amino acids of the protein, resulting in an expression construct (pAMC1) encoding an 856 amino acid protein (SLC4A11 v2-M36), was created using the Q5^®^ site directed mutagenesis kit. The primers used were 5′atctcgcagaatggatacttcg3′ and 5′gctagccagcttgggtct3′. gBlock gene fragment encoding the first 135 amino acids of SLC4A11 variant 1 with a BamHI site at the 5′ end was synthesized from Integrated DNA technologies and cloned blunt in pJET1.2 vector (pDM16) using the CloneJET PCR cloning kit. This was used as a positive control for variant 1 amplification as it contains the target sequence amplified by SLC4A11 variant 1 primers. SLC4A11 variant 3 with a c-terminal HA epitope tag was in pCDNA3.1 (pCML13). Integrity of all the clones was confirmed by DNA sequencing (Institute for Biomolecular Design, Department of Biochemistry, University of Alberta).

### Bioinformatic analysis

SLC4A11 v2-M1 nucleotide sequences immediately upstream of the first and second possible translation initiation start codons (ATG) were compared to the human Kozak consensus sequence (GCCACC). To identify SLC4A11 genes in other organisms human SLC4A11 sequence was used as a BLAST query of other species genome databases available through Ensembl. Genomic DNA surrounding and inclusive of SLC4A11 coding sequence was exported and used to search for DNA sequences corresponding to the unique 297 bp, 184 bp and 147 bp regions of human SLC4A11 v1, 2, and 3, respectively. Genomic DNA was manually searched since many databases only contain predicted transcripts, not fully sequenced cDNAs. High sequence conservation allowed identification of the M36 codon readily. Sequences upstream of the M36 codon were carefully examined for the presence of an upstream M1 start codon corresponding to human v2-M1. Translated upstream sequences were aligned to human SLC4A11-v2-M1 amino acid sequences.

### Preparation of anti-SLC4A11 antibodies

Three polyclonal antibodies were raised in rabbits against synthetic peptides, corresponding to regions of human SLC4A11, by PrimmBiotech (Cambridge, MA USA). Antibody “SLC4A11-v2-M1” corresponded to amino acids 11–26 of variant 2, starting at the presumed start Met1, CTQEVQGLVHGAGDLS. Antibody “SLC4A11-common” corresponded to amino acids 37–50 of variant 2, starting at M36, SQNGYFEDSSYYKC. Antibody “SLC4A11-v3” was generated against the N-terminal residues of the unique exon in SLC4A11 variant 3, MAAATRRVFHLQPC.

### Cell culture and transfection

HEK293 cells were grown at 37 °C or 30 °C in 5% CO_2_/air environment in complete DMEM (DMEM, supplemented with 5% (v/v) fetal bovine serum, 5% (v/v) calf serum, and 1% (v/v) penicillin-streptomycin-glutamine. All experiments involving transiently transfected cells were carried out 40 to 48 h post-transfection. cDNA encoding SLC4A11 variants and eGFP were transiently transfected, using the calcium phosphate method^54^.

### Preparation of human corneal lysates

Human cadaver corneas, obtained post-mortem with informed consent of next of the kin of deceased donors, were prepared for transplant by the Comprehensive Tissue Centre (University of Alberta Hospital, Edmonton Canada). Human corneas used in this study were deemed unsuitable for transplant and made available for research. Experiments using human corneas were approved by the University of Alberta Human Research Ethics Board (HREB) and experiments followed guidelines from HREB. For protein isolation, six corneal samples were pooled. Average age of donors was 54 years and average endothelial cell density was 2641 cells/mm^2^. Lysates from intact corneas and endothelial layer were prepared separately. Briefly, intact corneas were frozen in liquid nitrogen and crushed using a mortar and pestle. The resulting tissue powder was solubilized in IP buffer (1% (v/v) IGEPAL CA-630, 5 mM EDTA, 150 mM NaCl, 0.5% (w/v) sodium deoxycholate, 10 mM Tris, pH 7.5), containing Complete Protease Inhibitor cocktail (mini-complete, Roche) and PMSF. For endothelial cell lysates, six corneas were micro-dissected and the endothelial layers were peeled off along with the Descemet’s membrane and pooled. Tissues were also lysed in IP buffer, containing Complete Protease Inhibitor cocktail. Both samples were incubated on ice for 20 min and were centrifuged at 13200 x g for 20 min at 4 °C and protein concentration was determined by BCA Assay^55^.

### Immunoblots

Cell lysates were prepared in 2X SDS-PAGE sample buffer (10% (v/v) glycerol, 2% (w/v) SDS, 0.5% (w/v) bromophenol blue, 75 mM Tris, pH 6.8), containing Complete Protease Inhibitor Cocktail (Roche). Lysates were made to 1% (v/v) 2-mercaptoethanol, heated for 5 min at 65 °C and insoluble material was removed by centrifugation at 16000 x g for 10 min. Samples were then resolved by SDS-PAGE on 7.5% (w/v) acrylamide gels^56^. Immunoblots were processed as described^34^. Mouse anti-GAPDH or mouse anti-GFP or Rabbit anti-SLC4A11-common or Rabbit anti-SLC4A11-v2-M1 or Rabbit anti-SLC4A11 v3 were used at 1:3000 or 1:3000 or 1:500 or 1:500 or 1:500 dilution in TBS-TM (5% skim milk powder in TBS-T: 0.1% (v/v) Tween-20, 0.15 M NaCl, 50 mM Tris-HCl, pH 7.5), respectively. After incubation with sheep anti-mouse or donkey anti-rabbit HRP conjugated secondary antibody at 1:5000 dilution, immunoblots were developed using Western Lightning™ Chemiluminescence Reagent Plus. Immunoblots containing corneal endothelial cell lysates incubated with Rabbit anti-SLC4A11-common or Rabbit anti-SLC4A11-variant 3 were developed using SuperSignal West Femto Maximum Sensitivity Substrate (Thermo Scientific). Immunoblots were visualized using ImageQuant LAS4000 (GE Healthcare). Quantitative densitometric analyses were performed, using ImageQuant TL 8.1 software.

### Cell surface processing assays

Cell surface processing assays were performed as described earlier^33^. Transfected cells were rinsed with 4 °C phosphate buffered saline (PBS) (140 mM NaCl, 3 mM KCl, 6.5 mM Na_2_HPO_3_, 1.5 mM KH_2_PO_3_, pH 7.4), washed with 4 °C Borate buffer (154 mM NaCl, 7.2 mM KCl, 1.8 mM CaCl_2_, 10 mM boric acid, pH 9.0) and labelled with Sulpho-NHS-SS-Biotin (0.5 mg/ml). After washing three times with 4 °C Quenching buffer (192 mM glycine, 25 mM Tris, pH 8.3), cells were solubilised in 500 µl of IPB buffer, containing Complete Protease Inhibitor. For each sample, half of the recovered supernatant was retained for later SDS-PAGE analysis (Total Protein, T). The remaining half of the recovered supernatants was combined with 100 µl of 50% suspension of High Capacity Streptavidin Agarose resin to precipitate the biotinylated proteins and the supernatant was collected (Unbound Protein, U). The T and U fractions of each sample were processed for SDS-PAGE analysis and immunoblotting as described above. Densitometry using GE image analysis Software and the formula (T-U)/T x 100% was used to calculate the percentage of biotinylated protein.

### Reverse transcription PCR

Human corneas, deemed unfit for transplantation, were from the Comprehensive Tissue Centre, University of Alberta Hospital. Corneas were either used intact or subjected to dissection to isolate corneal endothelium with Descemet’s membrane. Total corneal RNA and isolated endothelial cell RNA each came from three corneas, respectively with average donor age 62 and 52 years and average endothelial cell density 3256 and 3046 cells/mm^2^. RNA was isolated from tissues using Trizol reagent (Life Technologies) according to the manufacturer’s instructions. The resulting RNA was then used as template with SuperScript^TM^ III Reverse Transcriptase (Invitrogen) (or without enzyme as a negative control) to generate cDNA according to manufacturer’s instructions. Resulting cDNA (100 ng) was used as template in PCR with Taq Polymerase along with the corresponding primers (see Supplementary Table S1). PCR was performed using an MJ Research Inc. PTC-100 thermal cycler. PCR conditions were: 2 min 95 °C, 40 cycles of (30 s at 95 °C, 30 s at 54 °C (variant 1, 2, 3 primers), 15 s at 72 °C) and 10 min at 72 °C. Resulting PCR products were visualized by agarose gel electrophoresis. To check the specificity of each primer pair, PCR was performed using the primer pairs for each variant with three synthetic templates (pDM16, pSKL1 and pCML13), using similar conditions as mentioned above. Resulting PCR products were visualized by agarose gel electrophoresis.

### Real Time quantitative PCR

cDNA from total cornea and isolated endothelium were used with primer pairs for variant 1, 2, 3 and GAPDH (see Supplementary Table S1) and iQ SYBR green super mix on Rotor Gene RG-3000 real time analysis system (Corbett life Science, San Francisco, CA, USA). Reactions were performed in 20 µl reaction mixture with cDNA (from 1 ng RNA), 1X SYBR green mix and 300 nM of forward and reverse primer. Reactions without reverse transcriptase enzyme samples were used to quantify the background and reactions without template were used as a negative control for each group. Primers for GAPDH were used as an internal control. PCR conditions were: 10 min for 95 °C, 40 cycles of (10 s at 95 °C, 15 s at 54 °C, 20 s at 72 °C). To quantify transcript abundance, a standard curve was constructed for each variant using a range of copies for each synthetic template (2.5 to 2.5 × 10^4^ copies) in the similar reaction conditions as above. Fluorescence was recorded at the end of each elongation step. After 40 cycles, a melting curve was generated by slowly increasing the temperature (0.1 °C/s) from 54 °C to 95 °C, while the fluorescence was measured. The threshold cycle (C_T_) was calculated manually and kept consistent throughout. A standard curve was constructed for each variant by plotting the Log_10_ of the copies against the C_T_ values. The C_T_ for the samples were normalized for GAPDH and plotted in the standard curve to estimate the number of copies of each transcript per reaction. The values for No RT control were subtracted for each variant in each group.

### Assays of osmotically driven water flux

HEK293 cells were grown on poly-L-lysine-coated 25 mm round glass coverslips and co-transfected with cytosolic enhanced green fluorescent protein (eGFP) (peGFP-C1 vector, Clontech, USA) and, or pcDNA 3.1 (empty vector) or the indicated SLC4A11 plasmid constructs in a 1:8 molar ratio^19^. After 48 h, coverslips were mounted in a 35 mm diameter Attofluor Cell Chamber (Molecular Probes) and washed with 1X PBS. During experiments, the chamber was perfused at 3.5 ml/min with isotonic MBSS buffer (90 mM NaCl, 5.4 mM KCl, 0.4 mM MgCl_2_, 0.4 mM MgSO_4_, 3.3 mM NaHCO_3_, 2 mM CaCl_2_, 5.5 mM glucose, 100 mM D-mannitol and 10 mM HEPES, pH 7.4, 300 mOsm/kg) and then with hypotonic (200 mOsm/kg) MBSS buffer, pH 7.4 (same composition as previous but lacking D-mannitol). The chamber was mounted on the stage of a Wave FX Spinning Disc Confocal Microscope (Quorum Technologies, Guelph, Canada), with a Yokogawa CSU10 scanning head. The microscope has a motorized XY stage with Piezo Focus Drive (ASI, MS-4000 XYZ Automated Stage) and a live cell environment chamber (Chamlide, Korea), set to 24 °C for the duration of the experiment. Acquisition was performed with a Hamamatsu C9100-13 Digital Camera (EM-CCD) and a 20X objective during excitation with laser (Spectral Applied Research, Richmond Hill, ON, Canada) at 491 nm. eGFP fluorescence, collected though a dichroic cube (Quorum Technologies, Guelph, Canada) at wavelengths 520–540 nm, was acquired at 1 point s^−1^ for 4–6 min. Quantitative image analysis was performed by selecting a region of interest for each HEK293 cell with Volocity 6.0 software (PerkinElmer, ON, Canada). Following the switch to hypotonic medium, the rate of fluorescence change was determined from the initial 15 s of linear fluorescence change.

### Quantification of SLC4A11 variant 2 and 3 proteins in endothelial cell lysates

HEK293 cells transfected with cDNA encoding SLC4A11 v2, v3 and empty vector were lysed as explained above. v2 lysate (10 µg protein), v3 lysates (5 µg, 10 µg and 20 µg protein), vector control lysate (20 µg protein) and pooled human corneal endothelial cell lysate (20 µg protein) were processed on immunoblots in duplicates. Blots were probed with SLC4A11-common antibody and SLC4A11-v3 antibody, respectively followed by Donkey anti-rabbit secondary antibody and developed. GAPDH, used as a loading control, was detected with monoclonal antibody against GAPDH from Santa Cruz Biotechnology and secondary antibody was sheep anti-mouse (GE Healthcare Bio-Sciences Corp. (Piscattaway, NJ, USA). Band intensities (B.I.) were determined by densitometry. The ratio of v3 detection by anti-common and anti-v3 antibodies was determined (see Supplementary Fig. S1). This detection ratio (anti-common/anti-v3) was used to calculate the abundance of v2 and v3 in endothelial lysates as follows: Total SLC4A11 expression (detected with anti-common antibody) = v2 + v3. To convert the abundance of v3 detected by anti-v3 to the amount detected with anti-common, v3 = B.I._α-v3_ × Detection ratio. So, v2 = Total SLC4A11 expression (detected with anti-common antibody) - v3 and thus v2 = Total SLC4A11 expression (detected with anti-common antibody) - (B.I._α-v3_ × Detection ratio).

### Homology modeling of SLC4A11 v2-M36 and v3 cytoplasmic domains

Cytoplasmic domain sequence of SLC4A11 v2-M36 (M36-Y340) and SLC4A11 v3 (M1-Y359) were used to prepare their respective homology models using Protein Homology/Analogy Recognition Engine V 2.0 (Phyre^2^)^51^ and Iterative Threading Assembly Refinement (I-TASSER)^50^. Crystal structure of human erythrocyte Band 3 (SLC4A1) cytoplasmic domain (PDB: 1HYN) was used as the template. Generated models were aligned in The PyMOL molecular graphics system v 2.0.7 using segments that were modelled with 100% confidence (R90-P306 for v2, R109-P325 for v3).

### Statistical analysis

Numerical values were represented as mean ± standard error of measurement (SEM). Statistical analyses were performed using GraphPad Prism 7.0. (GraphPad software). Groups were compared with one-way and two-way ANOVA with Sidak-post tests, and two-tailed t-tests. p < 0.05 was considered significant.

## Supplementary information

Supplementary Figures S1-S5 and Supplementary Tables S1 and S2

## Data Availability

The datasets generated during and/or analysed during the current study are available from the corresponding author on reasonable request.

## References

[1] Klintworth GK (2009). Corneal dystrophies. Orphanet. J. Rare Dis..

[2] Bonanno JA (2003). Identity and regulation of ion transport mechanisms in the corneal endothelium. Prog Retin Eye Res.

[3] 2016 Eye Banking Statisitical Report. (Eye Bank Association of America, Washington, D.C., U.S.A., 2017).

[4] Vithana EN (2008). SLC4A11 Mutations in Fuchs Endothelial Corneal Dystrophy (FECD). Hum. Mol. Genet..

[5] Biswas S (2001). Missense mutations in COL8A2, the gene encoding the alpha2 chain of type VIII collagen, cause two forms of corneal endothelial dystrophy. Hum. Mol. Genet..

[6] Riazuddin SA (2010). Missense mutations in TCF8 cause late-onset Fuchs corneal dystrophy and interact with FCD4 on chromosome 9p. Am. J. Hum. Genet..

[7] Riazuddin SA, Vasanth S, Katsanis N, Gottsch JD (2013). Mutations in AGBL1 cause dominant late-onset Fuchs corneal dystrophy and alter protein-protein interaction with TCF4. Am. J. Hum. Genet..

[8] Riazuddin SA (2012). Mutations in LOXHD1, a recessive-deafness locus, cause dominant late-onset Fuchs corneal dystrophy. Am. J. Hum. Genet..

[9] Afshari NA (2017). Genome-wide association study identifies three novel loci in Fuchs endothelial corneal dystrophy. Nat Commun.

[10] Baratz KH (2010). E2-2 protein and Fuchs’s corneal dystrophy. N Engl J Med.

[11] Hemadevi B (2008). Identification of mutations in the SLC4A11 gene in patients with recessive congenital hereditary endothelial dystrophy. Arch Ophthalmol.

[12] Jiao X (2007). Autosomal recessive corneal endothelial dystrophy (CHED2) is associated with mutations in SLC4A11. J Med Genet.

[13] Ramprasad VL (2007). Novel SLC4A11 mutations in patients with recessive congenital hereditary endothelial dystrophy (CHED2). Hum. Mutat..

[14] Sultana, A., Garg, P., Ramamurthy, B., Vemuganti, G. K. & Kannabiran, C. Mutational spectrum of the SLC4A11 gene in autosomal recessive congenital hereditary endothelial dystrophy. *Mol Vis* **13**, 1327–1332; v13/a145 [pii] (2007). 17679935

[15] Vithana EN (2006). Mutations in sodium-borate cotransporter SLC4A11 cause recessive congenital hereditary endothelial dystrophy (CHED2). Nature Genet..

[16] Patel SP, Parker MD (2015). SLC4A11 and the Pathophysiology of Congenital Hereditary Endothelial Dystrophy. BioMed Res. Intl..

[17] Desir J (2007). Borate transporter SLC4A11 mutations cause both Harboyan syndrome and non-syndromic corneal endothelial dystrophy. J. Med. Genet..

[18] Siddiqui S (2014). Congenital hereditary endothelial dystrophy caused by SLC4A11 mutations progresses to Harboyan syndrome. Cornea.

[19] Vilas GL (2013). Transmembrane water-flux through SLC4A11: a route defective in genetic corneal diseases. Hum Mol Genet.

[20] Loganathan SK, Casey JR (2014). Corneal dystrophy-causing SLC4A11 mutants: suitability for folding-correction therapy. Human Mutat..

[21] Jalimarada SS, Ogando DG, Vithana EN, Bonanno JA (2013). Ion transport function of SLC4A11 in corneal endothelium. Invest. Ophthalmol. Vis. Sci..

[22] Ogando DG, Jalimarada SS, Zhang W, Vithana EN, Bonanno JA (2013). SLC4A11 is an EIPA-sensitive Na^+^ permeable pHi regulator. Am. J. Physiol. Cell. Physiol..

[23] Kao L, Azimov R, Abuladze N, Newman D, Kurtz I (2015). Human SLC4A11-C functions as a DIDS-stimulatable H^+^(OH^−^) permeation pathway: partial correction of R109H mutant transport. Am. J. Physiol. Cell. Physiol..

[24] Myers EJ, Marshall A, Jennings ML, Parker MD (2016). Mouse Slc4a11 expressed in *Xenopus* oocytes is an ideally selective H^+^/OH^−^ conductance pathway that is stimulated by rises in intracellular and extracellular pH. Am. J. Physiol. Cell. Physiol..

[25] Zhang W, Ogando DG, Bonanno JA, Obukhov AG (2015). Human SLC4A11 Is a Novel NH_3_/H^+^ Co-transporter. J. Biol. Chem..

[26] Loganathan SK, Schneider HP, Morgan PE, Deitmer JW, Casey JR (2016). Functional Assessment of SLC4A11, an Integral Membrane Protein Mutated in Corneal Dystrophies. Am. J. Physiol..

[27] Jennings ML, Howren TR, Cui J, Winters MJ, Hannigan R (2007). Transport And Regulatory Characteristics Of The Yeast Bicarbonate Transporter Homolog Bor1p. Am. J. Physiol..

[28] Park M, Li Q, Shcheynikov N, Zeng W, Muallem S (2004). NaBC1 is a ubiquitous electrogenic Na^+^ -coupled borate transporter essential for cellular boron homeostasis and cell growth and proliferation. Mol. Cell.

[29] Alka K, Casey JR (2018). Molecular Phenotype of SLC4A11 Missense Mutants: Setting the Stage for Personalized Medicine in Corneal Dystrophies. Human Mutat..

[30] Li S (2018). R125H, W240S, C386R, and V507I SLC4A11 mutations associated with corneal endothelial dystrophy affect the transporter function but not trafficking in PS120 cells. Exp.Eye Res..

[31] Loganathan SK, Lukowski CM, Casey JR (2016). The cytoplasmic domain is essential for transport function of the integral membrane transport protein SLC4A11. Am. J. Physiol..

[32] Soumittra N (2014). Biosynthetic and functional defects in newly identified SLC4A11 mutants and absence of COL8A2 mutations in Fuchs endothelial corneal dystrophy. J Hum Genet.

[33] Vilas GL (2012). Oligomerization of SLC4A11 protein and the severity of FECD and CHED2 corneal dystrophies caused by SLC4A11 mutations. Human Mutat..

[34] Vilas GL, Morgan PE, Loganathan S, Quon A, Casey JR (2011). A Biochemical Framework for SLC4A11, the Plasma Membrane Protein Defective in Corneal Dystrophies. Biochem.

[35] Puangsricharern V, Yeetong P, Charumalai C, Suphapeetiporn K, Shotelersuk V (2014). Two novel mutations including a large deletion of the SLC4A11 gene causing autosomal recessive hereditary endothelial dystrophy. Br. J. Ophthalmol..

[36] Kodaganur SG (2013). Mutation analysis of the SLC4A11 gene in Indian families with congenital hereditary endothelial dystrophy 2 and a review of the literature. Mol Vis.

[37] Aldahmesh M, Khan A, Meyer B, Alkuraya F (2009). Mutational Spectrum of SLC4A11 in Autosomal Recessive CHED in Saudi Arabia. Invest. Ophthalmol. Vis. Sci..

[38] Aldave AJ (2007). Autosomal Recessive CHED Associated With Novel Compound Heterozygous Mutations in SLC4A11. Cornea.

[39] Kumar A, Bhattacharjee S, Prakash DR, Sadanand CS (2007). Genetic analysis of two Indian families affected with congenital hereditary endothelial dystrophy: two novel mutations in SLC4A11. Mol Vis.

[40] Kozak M (1986). Point mutations define a sequence flanking the AUG initiator codon that modulates translation by eukaryotic ribosomes. Cell.

[41] Parker MD, Ourmozdi EP, Tanner MJ (2001). Human BTR1, a New Bicarbonate Transporter Superfamily Member and Human AE4 from Kidney. Biochem. Biophys. Res. Commun..

[42] Riazuddin SA (2010). Missense mutations in the sodium borate co-transporter SLC4A11 cause late onset Fuchs corneal dystrophy. Hum. Mutat..

[43] Roy S, Praneetha DC, Vendra VP (2015). Mutations in the Corneal Endothelial Dystrophy-Associated Gene SLC4A11 Render the Cells More Vulnerable to Oxidative Insults. Cornea.

[44] Kao L (2016). Multifunctional ion transport properties of human SLC4A11: comparison of the SLC4A11-B and SLC4A11-C variants. Am. J. Physiol. Cell. Physiol..

[45] Guha S, Chaurasia S, Ramachandran C, Roy S (2017). SLC4A11 depletion impairs NRF2 mediated antioxidant signaling and increases reactive oxygen species in human corneal endothelial cells during oxidative stress. Sci. Rep..

[46] Chiu AM, Mandziuk JJ, Loganathan SK, Alka K, Casey JR (2015). High Throughput Assay Identifies Glafenine as a Corrector for the Folding Defect in Corneal Dystrophy-Causing Mutants of SLC4A11. Invest. Ophthalmol. Vis. Sci..

[47] Badior KE, Alka K, Casey JR (2017). SLC4A11 Three-Dimensional Homology Model Rationalizes Corneal Dystrophy-Causing Mutations. Hum. Mutat..

[48] Alka K, Casey JR (2018). Ophthalmic Nonsteroidal Anti-Inflammatory Drugs as a Therapy for Corneal Dystrophies Caused by SLC4A11 Mutation. Invest. Ophthalmol. Vis. Sci..

[49] Arakawa T (2015). Crystal structure of the anion exchanger domain of human erythrocyte band 3. Science (New York, N.Y.).

[50] Yang J (2015). The I-TASSER Suite: protein structure and function prediction. Nat Methods.

[51] Kelley LA, Mezulis S, Yates CM, Wass MN, Sternberg MJ (2015). The Phyre^2^ web portal for protein modeling, prediction and analysis. Nat Protoc.

[52] Blom N, Gammeltoft S, Brunak S (1999). Sequence and structure-based prediction of eukaryotic protein phosphorylation sites. J Mol Biol.

[53] Graham MA, Rawe I, Dartt DA, Joyce NC (2000). Protein kinase C regulation of corneal endothelial cell proliferation and cell cycle. Invest. Ophthalmol. Vis. Sci..

[54] Ruetz S, Lindsey AE, Ward CL, Kopito RR (1993). Functional activation of plasma membrane anion exchangers occurs in a pre-Golgi compartment. J. Cell Biol..

[55] Smith PK (1985). Measurement of protein using bicinchoninic acid. Anal. Biochem..

[56] Laemmli UK (1970). Cleavage of structural proteins during assembly of the head of bacteriophage T4. Nature.

