# Supplementary material for: Human Corneal Expression of SLC4A11, a Gene Mutated in Endothelial Corneal Dystrophies
Source: Sci Rep. 2019 Jul 4;9:9681. doi: 10.1038/s41598-019-46094-y (PMC6609610; doi:10.1038/s41598-019-46094-y)
Supplement: Supplementary file 1 — Supplementary Figures S1-S5 and Supplementary Tables S1 and S2 [file 41598_2019_46094_MOESM1_ESM.pdf]

Supplementary Information

Human Corneal Expression of SLC4A11, a Gene Mutated in Endothelial Corneal Dystrophies

Darpan Malhotra[1], Sampath K. Loganathan[1,2], Anthony M. Chiu[1], Chris M. Lukowski[1] and Joseph R. Casey[1#]

[1]Department of Biochemistry, Membrane Protein Disease Research Group, University of Alberta, Edmonton, Alberta, T6G 2H7, Canada.

[2]Present address: Lunenfeld-Tanenbaum Research Institute, Toronto, Ontario, Canada M5G 1X5

[#]Address Correspondence to:
Dr. Joseph R. Casey
Department of Biochemistry
University of Alberta
Edmonton, Alberta, Canada T6G 2H7
Phone: (780) 492-7203
Email: joe.casey@ualberta.ca
URL: http://www2.biochem.ualberta.ca/CaseyLab/index.php

SUPPLEMENTARY FIGURE LEGENDS

Supplementary Figure S1. Detection ratio of SLC4A11-common antibody vs. SLC4A11-v3 antibody for SLC4A11 v3 protein. HEK293 cells transfected with cDNA encoding SLC4A11 v3 were processed for immunoblots. 5 μg, 10 μg and 20 μg of cell lysate was loaded in duplicates. Blots were probed with SLC4A11-common antibody (α-common) and SLC4A11-v3 (α-v3) antibody. Densitometry quantified the band intensities. Ratio of SLC4A11 band intensity from α–common and α–v3 antibodies was calculated for each amount of total lysate and plotted. Data represent mean ± SEM from three replicates.

Supplementary Figure S2. Alternative homology model of SLC4A11 v2-M36 and SLC4A11 v3 cytoplasmic domains. Homology models of cytoplasmic domains of (**A**) SLC4A11 v2-M36 and (**B**) SLC4A11 v3 generated by Phyre2 fold recognition software, using crystal structure of human erythrocyte Band 3 cytoplasmic domain (PDB: 1HYN) as the template. Unique N-terminal sequence of v3 is highlighted in pink. (**C**) v2-M36 and v3 models aligned in PyMOL (molecular graphics system version 2.0.7), using segments that were modelled with 100% confidence (R90-P306 for v2-M36, R109-P325 for v3). N_{v2}, N_{v3} and C_{v2}, C_{v3} are the N and C termini for v2-M36 and v3, respectively.

Supplementary Figure S3. Full-length immunoblot images for the data presented in Figure 3. Blots 1 and 2 were probed with α-SLC4A11-common and α-SLC4A11-v3 antibodies, respectively. Blots probed with α-GAPDH antibody are presented below their

respective samples. Intervening lanes to the left of human cornea lane are shown. MW: Molecular weight.

Supplementary Figure S4. Full-length immunoblot images for the data presented in Figure 5A.

Supplementary Figure S5. Full-length immunoblot images for the data presented in Figure 6A.

SUPPLEMENTARY TABLES

Supplementary Table S1. Primers for reverse transcription PCR amplification of SLC4A11 transcripts.

	Forward Primer	Reverse Primer	Product Size (bp)
SLC4A11 Variant 1	5'-GGGCAGGGTTTTCTCAGGAA-3'	5'-CATTCTCAGTGTTGGTGGCCT-3'	244
SLC4A11 Variant 2	5'-GGCTGGTGACCTTTCTGCTT-3'	5'-CATTCTCAGTGTTGGTGGCCT-3'	233
SLC4A11 Variant 3	5'-CAGGCGCGTGTTCCATCTG-3'	5'-CATTCTCAGTGTTGGTGGCCT-3'	233
GAPDH	5'-CAGCCTCAAGATCATCAGCA-3'	5'-TGTGGTCATGAGTCCTTCCA-3'	106

Supplementary Table S2. Review of Human SLC4A11 Variants used in studies from 2001-2019. Studies using different SLC4A11 variants since 2001. Indicated citations can be found in the main references.

SLC4A11 Variant used	Literature Citations with Reference Numbers in Manuscript
v1	Zhang *et al.* (2015)[20]
v2-M1	Parker *et al.* (2001)[44], Park *et al.* (2004)[23], Vithana *et al.* (2006)[10], Riazuddin *et al.* (2010)[5], Vilas *et al.* (2011)[29], Vilas *et al.* (2012)[28], Jalimarada *et al.* (2013)[16], Vilas *et al.* (2013)[14], Ogando *et al.* (2013)[17], Loganathan *et al.* (2014)[15], Soumittra *et al.* (2014)[27], Roy *et al.* (2015)[45], Loganathan *et al.* (2016)[26], Kao *et al.* (2016)[46], Guha *et al.* (2017)[47], Li *et al.* (2019)[25]
v2-M36	Chiu *et al.* (2016)[48], Loganathan *et al.* (2016)[21], Badior *et al.* (2017)[49], Alka *et al.* (2018a)[24], Alka *et al.* (2018b)[50]
v3	Kao *et al.* (2015)[18], Kao *et al.* (2016)[46]

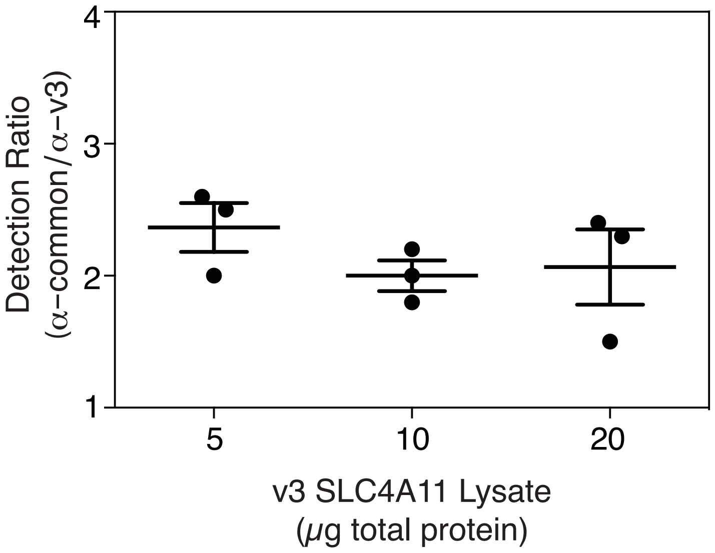

Supplementary Figure S1

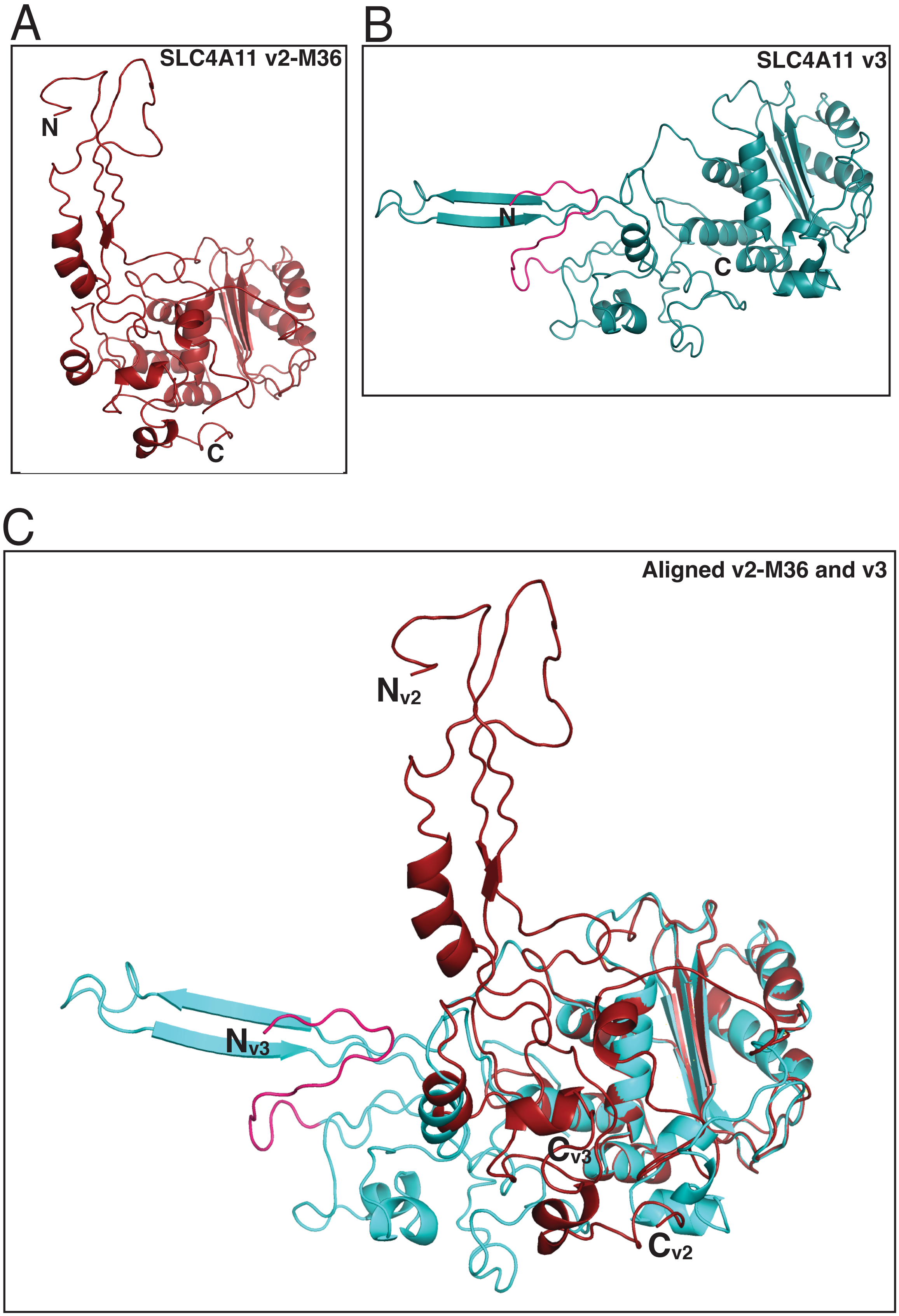

Supplementary Figure S2

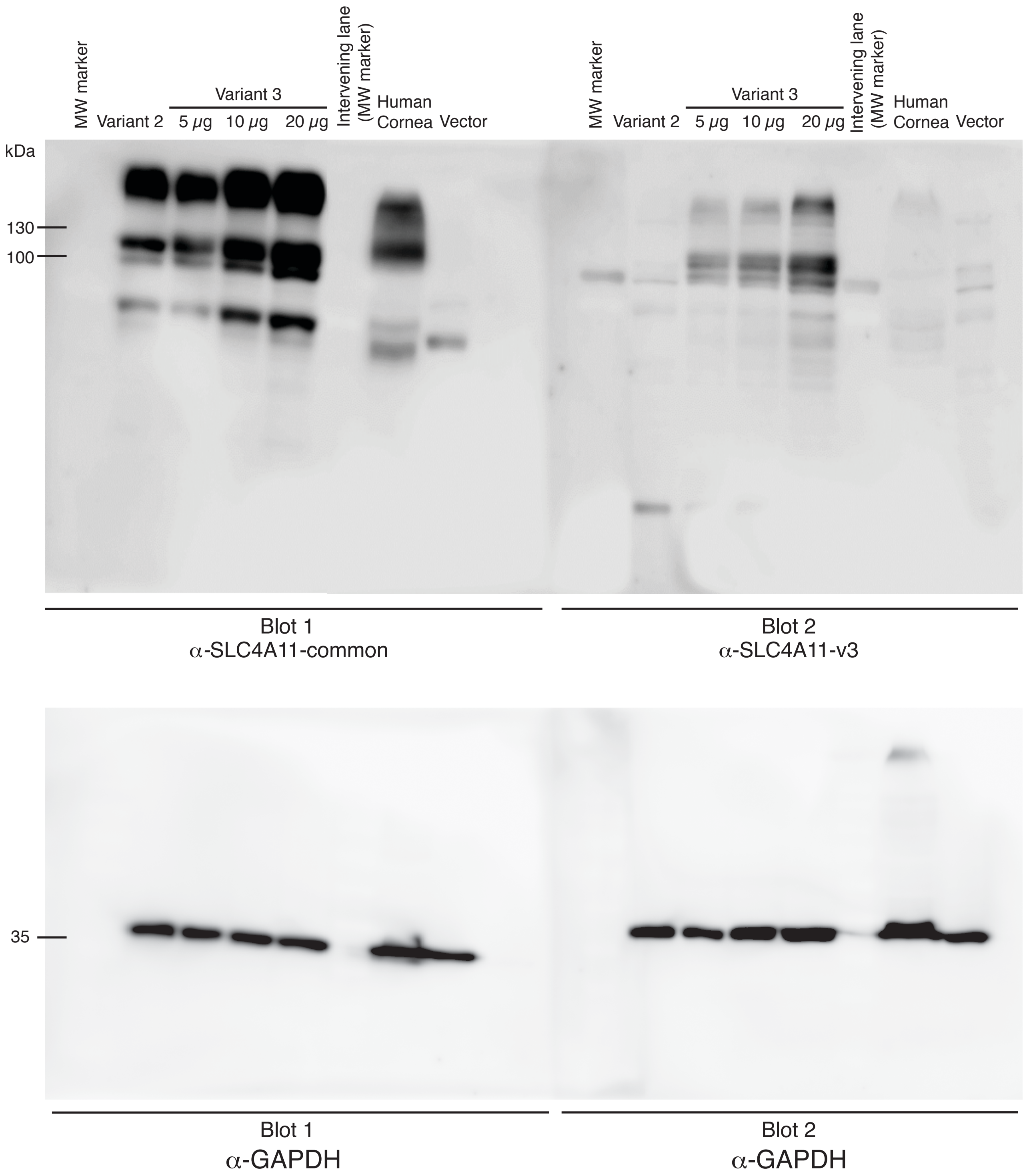

Supplementary Figure S3

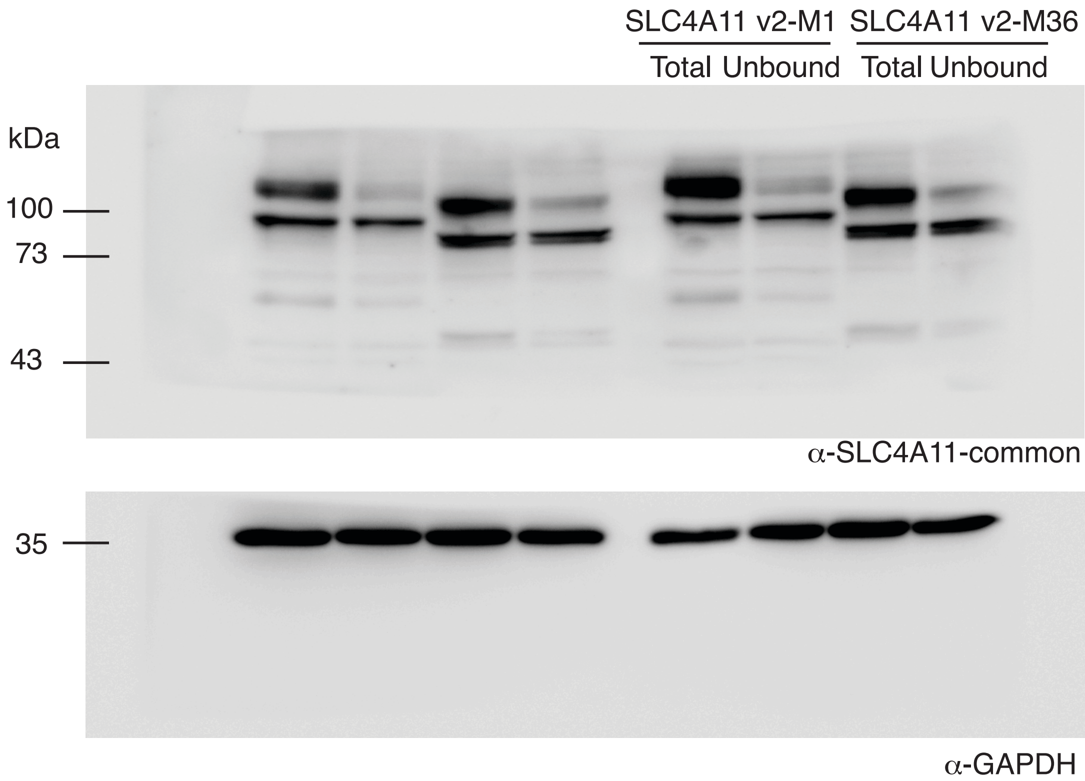

Supplementary Figure S4

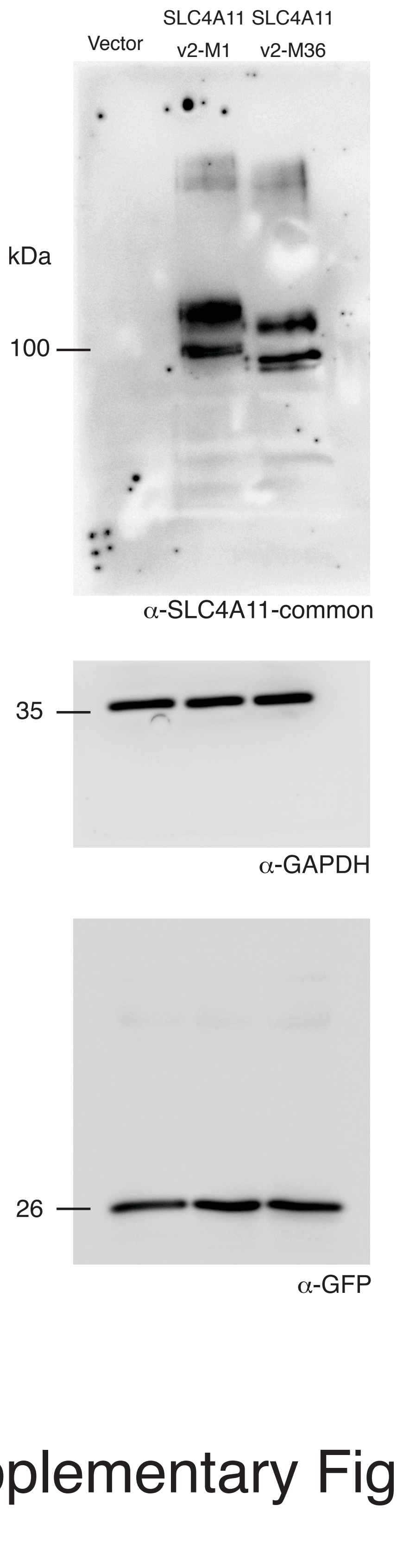

Supplementary Figure S5
